# Orally active microtubule-targeting agent, MPT0B271, for the treatment of human non-small cell lung cancer, alone and in combination with erlotinib

**DOI:** 10.1038/cddis.2014.128

**Published:** 2014-04-10

**Authors:** A-C Tsai, C-Y Wang, J-P Liou, H-C Pai, C-J Hsiao, J-Y Chang, J-C Wang, C-M Teng, S-L Pan

**Affiliations:** 1 School of Pharmacy, College of Pharmacy, Taipei Medical University, No. 250, Wu-hsing Street, Taipei 11031, Taiwan; 2 Pharmacological Institute, College of Medicine, National Taiwan University, Taipei, Taiwan; 3 National Institute of Cancer Research, National Health Research Institutes, Tainan, Taiwan; 4 Division of Hematology and Oncology, Department of Internal Medicine, National Cheng Kung University Hospital, Tainan, Taiwan; 5 The Ph.D. Program for Cancer Biology and Drug Discovery, College of Medical Science and Technology, Taipei Medical University, No. 250, Wu-hsing Street, Taipei 11031, Taiwan

**Keywords:** non-small cell lung cancer, microtubule-binding agents, apoptosis, erlotinib

## Abstract

Microtubule-binding agents, such as taxanes and vinca alkaloids, are used in the treatment of cancer. The limitations of these treatments, such as resistance to therapy and the need for intravenous administration, have encouraged the development of new agents. MPT0B271 (N-[1-(4-Methoxy-benzenesulfonyl)-2,3-dihydro-1H-indol-7-yl]-1-oxy-isonicotinamide), an orally active microtubule-targeting agent, is a completely synthetic compound that possesses potent anticancer effects *in vitro* and *in vivo*. Tubulin polymerization assay and immunofluorescence experiment showed that MPT0B271 caused depolymerization of tubulin at both molecular and cellular levels. MPT0B271 reduced cell growth and viability at nanomolar concentrations in numerous cancer cell lines, including a multidrug-resistant cancer cell line NCI/ADR-RES. Further studies indicated that MPT0B271 is not a substrate of P-glycoprotein (P-gp), as determined by flow cytometric analysis of rhodamine-123 (Rh-123) dye efflux and the calcein acetoxymethyl ester (calcein AM) assay. MPT0B271 also caused G2/M cell-cycle arrest, accompanied by the up-regulation of cyclin B1, p-Thr161 Cdc2/p34, serine/threonine kinases polo-like kinase 1, aurora kinase A and B and the downregulation of Cdc25C and p-Tyr15 Cdc2/p34 protein levels. The appearance of MPM2 and the nuclear translocation of cyclin B1 denoted M phase arrest in MPT0B271-treated cells. Moreover, MPT0B271 induced cell apoptosis in a concentration-dependent manner; it also reduced the expression of Bcl-2, Bcl-xL, and Mcl-1 and increased the cleavage of caspase-3 and -7 and poly (ADP-ribose) polymerase (PARP). Finally, this study demonstrated that MPT0B271 in combination with erlotinib significantly inhibits the growth of the human non-small cell lung cancer A549 cells as compared with erlotinib treatment alone, both *in vitro* and *in vivo*. These findings identify MPT0B271 as a promising new tubulin-binding compound for the treatment of various cancers.

Microtubules are the main component of the cytoskeleton and have an essential role in cell division, intracellular transport, and motility.^1^ Several clinically important microtubule-targeting agents, such as taxanes and vinca alkaloids, bind to microtubules, thereby altering the normal dynamic equilibrium and resulting in either the stabilization or destabilization of microtubules.^2^ The consequence of disrupting the microtubule organization with these drugs is the G2/M-phase arrest of the cell cycle and ultimately apoptotic or non-apoptotic cell death.^3, 4^

Even though microtubule-targeting agents are used clinically to treat patients with neoplastic disease, they have substantial drawbacks such as the development of resistance over time.^5^ Drug resistance can be intrinsic, in which case the first chemotherapy fails, or acquired, in which case there is a response to the first round of chemotherapy but failure of the second.^6^ In either case, tumors become refractory to various structurally different compounds. The most common form of drug resistance to taxanes and other microtubule-targeting agents is overexpression of the efflux pump P-glycoprotein (P-gp)/multidrug resistance (MDR) protein.^7^ Clinically used microtubule-targeting agents, such as paclitaxel and vinblastine, are substrates for P-gp. Thus, a microtubule-targeting agent is required, which circumvents these mechanisms of drug resistance. Such a treatment could be advantageous for patients with drug-resistant tumors.^8, 9, 10^ Furthermore, another limitation of current treatments is their requirement for intravenous delivery, which leads to hypersensitivity reactions.^11, 12^

Signal transducer and activator of transcription 3 (STAT3) is inappropriately activated in various tumor types, such as lymphoma, breast, ovarian, pancreatic, and lung cancer, and is particularly activated in aggressive and invasive tumors.^13, 14^ STAT3 is a transcription factor that translocates into the nucleus, binding to its responsive element after activation. STAT3 regulates the expression of various genes including Bcl-xL, Mcl-1, cyclin D1, and p53, which regulate cell-cycle progression, cell survival, and proliferation.^15, 16^ In addition to its transcriptional function, STAT3 associates with the cytoskeleton and may thus regulate microtubule function. Microtubule-interfering agents, such as paclitaxel and vinorelbine, can inhibit STAT3 tyrosine phosphorylation.^17, 18^

Epidermal growth factor receptor (EGFR), a transmembrane glycoprotein, is frequently overexpressed in human tumors such as breast, ovarian, prostate, pancreatic, and non-small cell lung cancer (NSCLC). This overexpression is correlated with poor prognosis and worse clinical outcome. Thus, targeted therapies directed at blocking EGFR function are attractive potential therapeutics for cancer.^19^ Erlotinib, a reversible EGFR tyrosine kinase inhibitor, was initially approved for treatment of patients with advanced NSCLC who had failed one chemotherapy regimen. Although clinical results have demonstrated that erlotinib monotherapy showed a survival benefit in comparison with gemcitabine for patients with NSCLC or pancreatic cancer, resistance to erlotinib reduces its efficacy.^20, 21^ The T790M point mutation in EGFR causes a conformational change at the ATP binding pocket, thus decreasing the affinity between erlotinib and EGFR and leading to acquired/secondary resistance.^22, 23^

In the present study, we demonstrate that MPT0B271 (N-[1-(4-methoxy-benzenesulfonyl)-2,3-dihydro-1H-indol-7-yl]-1-oxy-isonicotinamide), an orally active compound, represses microtubule polymerization. We evaluated the antiproliferative activity of MPT0B271 against several cancer cell lines as well as P-gp-overexpressing NCI/ADR-RES cells. We investigated the action mechanism of MPT0B271 on cell-cycle progression and apoptosis. Furthermore, we explored the anticancer activity of MPT0B271 alone and in combination with erlotinib *in vitro and in vivo*. Our results suggest that MPT0B271 is a promising therapeutic candidate for the treatment of human cancers.

## Results

### MPT0B271 inhibits tubulin polymerization

**Figure 1:**
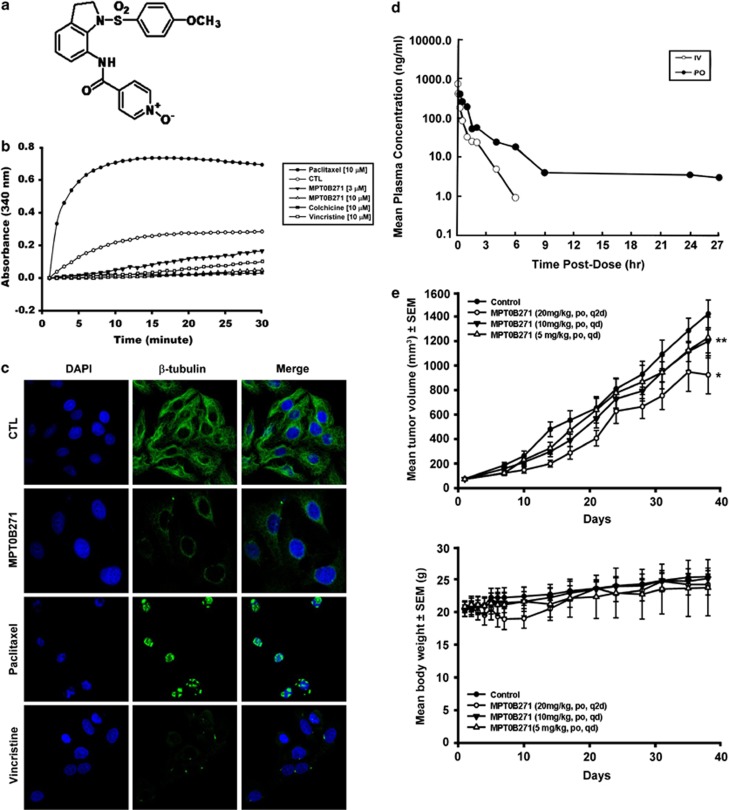
(**a**) Chemical structure of MPT0B271. (**b**) Effect of MPT0B271 on tubulin polymerization. Purified tubulin in reaction buffer was incubated at 37°C in the absence or presence of increasing concentrations of MPT0B271, 10 *μ*M paclitaxel, 10 *μ*M colchicine, or 10 *μ*M vincristine. Assembly of microtubules was then measured over 30 min at 1 min intervals at an absorbance of 340 nm using a spectrophotometer. (**c**) Immunofluorescence staining of microtubules in A549 cells. Cells were treated with vehicle (DMSO), 0.3 *μ*M MPT0B271, paclitaxel, or vincristine for 24 h. Cells were labeled with a *β*-tubulin antibody and an FITC-conjugated anti-mouse IgG antibody, were counterstained with 4,6-diamidino-2-phenylindole (DAPI) and observed by confocal microscopy. *Left*, DAPI; *middle*, microtubule network; *right*, merged microtubule network and DAPI. (**d**) PK properties, plasma concentration *versus* time profiles of MPT0B271 after i.v. (2 mg/kg) and p.o. (20 mg/kg) dosing of fasted male CD-1 (*Crl.*) mice. (**e**) Efficacy of MPT0B271, dosed orally, on tumor xenografts. *Upper panel*, tumor growth of A549 xenografts in nude mice that were orally treated with or without MPT0B271 (5, 10, and 20 mg/kg). Tumor growth is presented as the mean tumor volume (mm^3^)±S.E. Tumor volume was determined using caliper measurements and was calculated as the product of 1/2 × length × width^2^. *Lower panel*, body weight (g) of the mice. *P<0.05 and **P<0.01 as compared with the control group

MPT0B271 (Figure 1a) inhibited microtubule formation in an *in vitro* tubulin polymerization assay, and the effect was similar to the inhibition induced by the microtubule-destabilizing agents colchicine and vincristine. In contrast, the widely used microtubule-stabilizing agent paclitaxel induced a marked increase in tubulin polymerization (Figure 1b). The effect of MPT0B271 on the arrangement and distribution of the microtubule network in cells was studied *in situ* by immunofluorescence. As shown in Figure 1c, treatment of A549 cells with MPT0B271 disrupted the microtubule cytoskeleton. These data indicate that MPT0B271 depolymerizes microtubules in tumor cells.

### MPT0B271 is an orally bioavailable synthetic inhibitor of tumor growth *in vivo*

The MPT0B271 plasma concentration *versus* time profile following intravenous and oral administration to CD-1 (*Crl.*) mice is shown in Figure 1d. The half-life (*t*_1/2_) for oral administration (2.0 h) was slightly longer than the half-life for intravenous administration with the absolute oral bioavailability being 26%. At an oral dose of 20 mg/kg, MPT0B271 showed rapid absorption in mice with a short maximal plasma concentration time (*T*_max_) of 15 min (the first sampling time point). The mean *C*_max_ and AUC_(0–27)_ of MPT0B271 was 402 mg/ml and 558 ng h/ml, respectively. To evaluate the effect of MPT0B271 *in vivo*, nude mice that received tumor xenografts of the human NSCLC cell line A549 were used. As illustrated in Figure 1e (upper panel), oral administration of MPT0B271 resulted in a dose-dependent inhibition of tumor growth, and animals did not lose >10% of their body weight during treatment (Figure 1e, lower panel).

### MPT0B271 inhibits cancer cell proliferation and induces cytotoxicity *in vitro*

**Figure 2:**
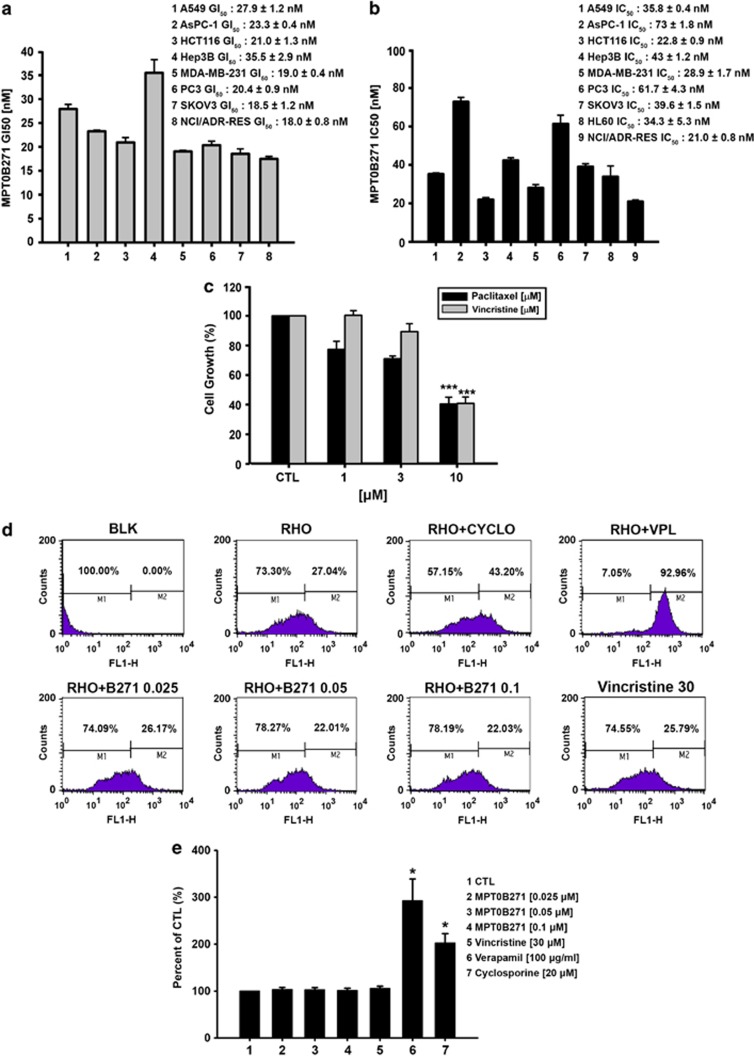
(**a**) Various types of human cancer cells were treated with the indicated concentrations of MPT0B271 for 48 h. Then, cell growth inhibition was determined using the SRB assay, and the GI_50_ of each cell line is expressed as the mean±S.E. of four independent determinations. (**b**) The cytotoxic effects of various human cancer cell lines were determined using an MTT assay. The IC_50_ of each cell line is expressed as the mean±S.E. of four independent determinations. (**c**) NCI/ADR-RES cells were treated with the indicated concentration of paclitaxel or vincristine for 48 h, and cell growth was determined by the SRB assay. Data are expressed as the mean±S.E. of at least three independent experiments. ***P<0.01 as compared with the control group. (**d**) Effect of MPT0B271 on P-gp activity. NCI/ADR-RES cells were pretreated with or without MPT0B271 (0.025, 0.05, and 0.1 *μ*M), verapamil (50 *μ*M), cyclosporine A (10 *μ*M), or vincristine (30 *μ*M) for 1 h and then co-treated with 10 *μ*M rhodamine 123 (Rh-123). After 1 h incubation at 37°C, cells were washed with PBS, collected by trypsinization and detected by flow cytometry. (**e**) NCI/ADR-RES cells were incubated in the absence or presence of the indicated agents for 30 min and then stained with calcein AM fluorescent dye. Fluorescence was measured at an excitation wavelength of 485 nm and an emission wavelength of 535 nm. *P<0.05 as compared with the control group

The ability of MPT0B271 to inhibit cancer cell growth was evaluated using the Sulforhodamine B (SRB) assay. As shown in Figure 2a, MPT0B271 inhibited the proliferation of A549, AsPC-1, HCT116, Hep3B, MDA-MB-231, PC3, SKOV3, and NCI/ADR-RES cells with GI_50_ values of 27.9, 23.3, 21.0, 35.5, 19.0, 20.4, 18.5, and 18 nM, respectively. MPT0B271 reduced the viability of several human cancer cell lines with IC_50_ values in the low nanomolar range of 21–73 nM, as measured by the 3-(4,5-dimethylthiazol-2-yl)-2,5-diphenyltetrazolium bromide (MTT) assay (Figure 2b).

### Effects of paclitaxel and vincristine on the growth of NCI/ADR-RES cells

MPT0B271 was equally potent against the multidrug-resistant cancer cell line NCI/ADR-RES, as measured by both SRB and MTT assays. The growth inhibition of NCI/ADR-RES cells treated with paclitaxel or vincristine was then evaluated. As shown in Figure 2c, the mean GI_50_ values of paclitaxel and vincristine were 7.670±1.228 *μ*M and 8.156±0.854 *μ*M, respectively, indicating that the effect of MPT0B271 was more potent than that of paclitaxel or vincristine in NCI/ADR-RES cells. Next, we performed flow cytometric analysis to measure the accumulation of rhodamine-123 (Rh-123), a P-gp-transported fluorescent dye, in NCI/ADR-RES cells following the treatment with MPT0B271. After incubation with classic P-gp inhibitors, verapamil and cyclosporine A, we observed a large increase in Rh-123 accumulation in comparison with the vehicle-treated cells. However, Rh-123 accumulation did not increase in NCI/ADR-RES cells after incubation with MPT0B271 (Figure 2d). Calcein acetoxymethyl ester (calcein AM) is a fluorescent substrate for P-gp and can therefore be used to measure the transport activity of P-gp. Thus, P-gp inhibition was examined by measuring calcein AM retention using the Multidrug Resistant Assay.^24^ As illustrated in Figure 2e, the increase in intracellular fluorescence was indicative of P-gp inhibition by verapamil or cyclosporine A. In contrast, the efflux of calcein AM was not affected by MPT0B271. These results suggest that MPT0B271 circumvents P-gp-mediated drug resistance.

### MPT0B271 arrests the cell cycle in M phase

**Figure 3:**
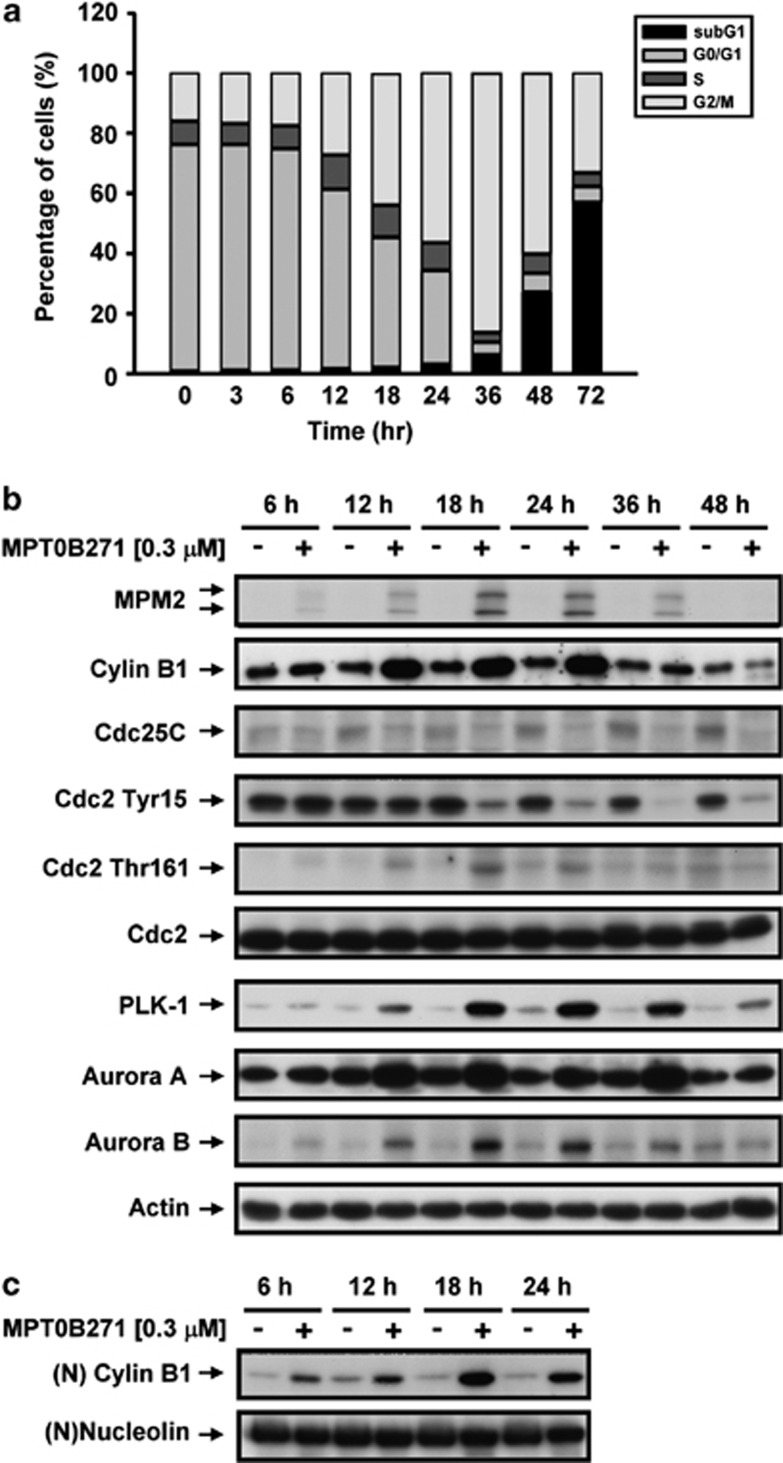
(**a**) Effect of MPT0B271 on cell-cycle progression. A549 cells were exposed to 0.3 *μ*M MPT0B271 for the indicated times and then stained with PI to determine the proportion of DNA. Data acquisition and analysis were performed on a FACScan flow cytometer. The data are expressed as the mean±S.E. of at least three independent experiments. (**b**) The effect of MPT0B271 on G2/M cell-cycle regulatory proteins. A549 cells were treated with 0.3 *μ*M MPT0B271 for the indicated times. Whole-cell extracts were subjected to SDS-PAGE and immunoblotted with the indicated antibodies. (**c**) Treatment with 0.3 *μ*M MPT0B271 for the indicated times. Nuclear lysates were subjected to western blot analysis using an antibody specific for cyclin B1

Most tubulin-targeting agents induce cell-cycle arrests;^4^ therefore, the effect of MPT0B271 on cell-cycle progression was assessed by FACScan flow cytometry using propidium iodide (PI) staining. The data showed that treatment of A549 cells with MPT0B271 for 6–72 h resulted in a time-dependent accumulation of cell populations at the G2/M phase followed by a subsequent increase in the hypodiploid sub-G1 cell population, which indicated apoptosis (Figure 3a). To elucidate the mechanisms through which MPT0B271 induced the G2/M-phase arrests, the levels of the G2/M phase regulatory proteins were assessed. Western blot analysis revealed that MPT0B271 induced upregulation of cyclin B1, p-Thr161 Cdc2/p34, serine/threonine kinases polo-like kinase 1 (PLK1), and aurora kinase A and B; downregulation of Cdc25C and p-Tyr15 Cdc2/p34 protein levels; and a marked increase in mitosis-specific MPM2 phosphoprotein expression (Figure 3b). In addition, a remarkable nuclear accumulation of cyclin B1 protein was demonstrated in MPT0B271-treated A549 cells as compared with untreated cells (Figure 3c).

### MPT0B271 triggers apoptosis in A549 cells

**Figure 4:**
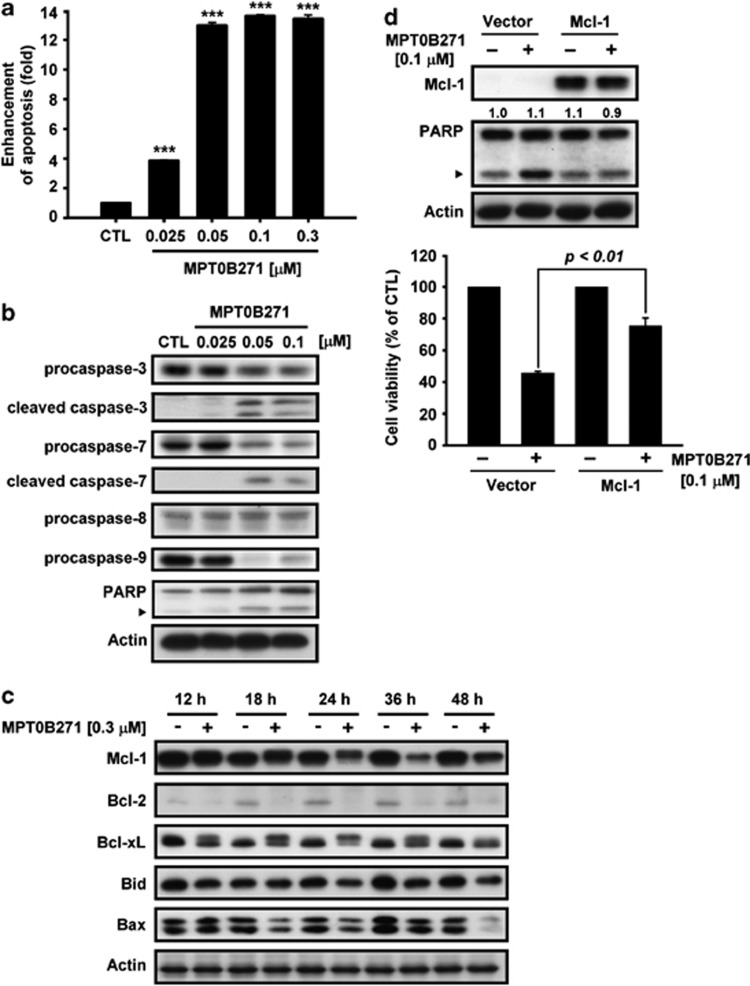
(**a**) Measurement of apoptosis. A549 cells were treated with the indicated concentration of MPT0B271, and oligonucleosomal DNA fragmentation was quantitatively assessed with the Cell Death ELISA^PLUS^ kit. Apoptosis was enhanced in relation to control cells. Data are expressed as the mean±S.E. of at least three independent experiments. ***P<0.001, as compared with the control group. (**b**) A549 cells were exposed to serial concentrations of MPT0B271 for 48 h, and whole-cell lysates were collected and immunoblotted with antibodies against caspase-3, -7, -8, and -9 and PARP. (**c**) After treatment with vehicle or MPT0B271 (0.3 *μ*M) for the indicated times, A549 cells were harvested and lysed. Equal amounts of lysate protein were run on an SDS-PAGE gel, transferred onto nitrocellulose membrane and incubated with the indicated antibodies. (**d**) Effect of ectopic Mcl-1 on MPT0B271-induced cell apoptosis. A549 cells were transfected with vector or Mcl-1 plasmid for 24 h and then incubated with or without MPT0B271 for 48 h. Whole-cell lysates were subjected to western blot analysis, and cell viability was measured by the SRB assay

The ability of MPT0B271 to induce apoptosis was examined by measuring cytoplasmic histone-associated DNA fragments using the Cell Death Detection ELISA^Plus^ kit. The data show that increasing concentrations of MPT0B271 increased the level of DNA fragments in A549 cells after 48 h of treatment (Figure 4a). Moreover, MPT0B271 caused a concentration-dependent increase in cleaved executioner caspase-3 and its downstream substrate poly (ADP-Ribose) polymerase (PARP) (Figure 4b). Based on these results, we investigated the effect of MPT0B271 on the expression of anti-apoptotic proteins. The protein levels of Bcl-2, Bcl-xL, and Mcl-1 in A549 cells decreased in a time-dependent manner after MPT0B271 treatment (Figure 4c). Furthermore, exogenous Mcl-1 overexpression partially abolished the activation of PARP cleavage and rescued cell viability in A549 cells, suggesting that MPT0B271 triggers apoptotic cell death through the suppression of Mcl-1 expression (Figure 4d).

### MPT0B271 inhibits constitutive p53 activation in A549 cells

**Figure 5:**
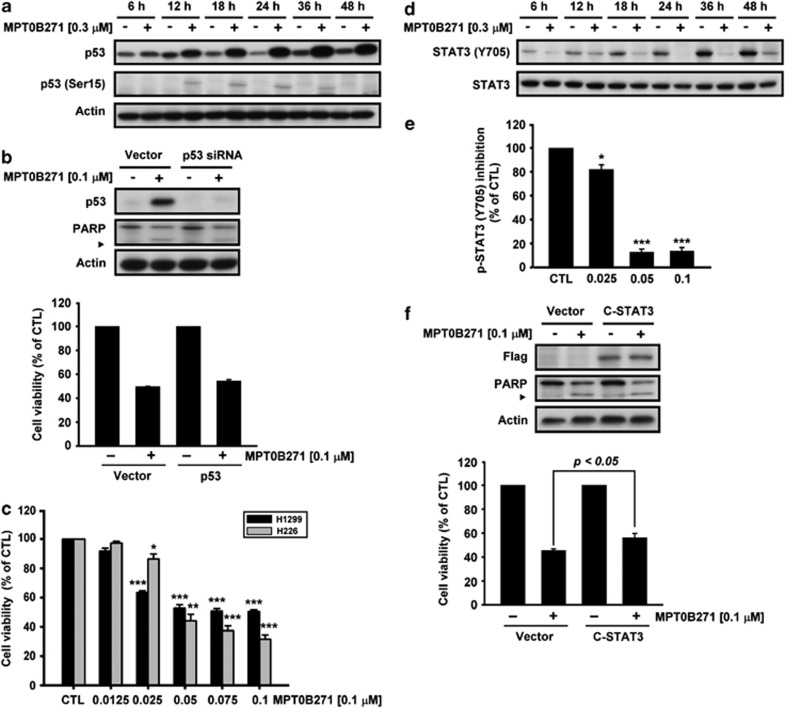
(**a**) The effect of MPT0B271 on p53 expression and phosphorylation in A549 cells. Cells were treated with MPT0B271 (0.3 *μ*M) for the indicated times, and whole-cell extracts were prepared and underwent western blot analysis using the indicated antibodies. (**b**) A549 cells were pretreated with or without p53 siRNA for 24 h and then incubated with or without MPT0B271 (0.1 *μ*M) for 48 h. *Upper panel*, total cellular lysates were subjected to western blot analysis of p53 and PARP. *Lower panel*, cell viability was measured by the SRB assay and expressed as a percentage of the untreated control. (**c**) Concentration-dependent effect of MPT0B271 on cell viability. H1299 and H226 cells were treated with or without the indicated concentration of MPT0B271 for 48 h, and the cytotoxic effect was determined with the MTT assay. Data are expressed as the mean±S.E. of at least three independent experiments. *P<0.05; **P<0.01, and ***P<0.001 as compared with the control group. (**d**) The effect of MPT0B271 on STAT3 phosphorylation in A549 cells. Cells were treated with MPT0B271 (0.3 *μ*M) for the indicated times, and whole-cell extracts were prepared and analyzed for STAT-3 phosphorylation (at Tyr^705^). (**e**) A549 cells were treated with various concentrations (0.025–0.1 *μ*M) of MPT0B271 for 24 h, after which the level of STAT3 tyrosine phosphorylation in cells was measured using the PathScan Phospho-Stat3 (Try705) Sandwich ELISA kit and spectrophotometry at 450 nm. Data represent the mean±S.E. of at least three independent experiments. *P<0.05; ***P<0.001 as compared with the control group. (**f**) The effect of ectopic STAT3 on MPT0B271-induced cell apoptosis. A549 cells were transfected with vector or STAT3 plasmid for 24 h and then incubated with or without MPT0B271 for 48 h. Whole-cell lysates were subjected to western blot analysis, and cell viability was measured by the SRB assay

The effect of MPT0B271 on p53 was evaluated by treating A549 cells for 6–48 h with MPT0B271 and then by immunoblotting with p53 and phospho-p53 (Ser15) antibodies. Treatment with MPT0B271 upregulated the expression of p53 protein and the phosphorylation of p53 at Ser15 (Figure 5a). However, knockdown of p53 by p53 small interfering RNA (siRNA) did not attenuate the induction of PARP and prevent the cytotoxic effects (Figure 5b). In addition, we further evaluated the *in vitro* cytotoxic activities of MPT0B271 in other human NSCLC cell lines, H1299 (null p53) and H226 (mutant p53), using the MTT assay. Treatment of H1299 and H226 cells with MPT0B271 reduced cell viability in a concentration-dependent manner, with mean IC_50_ values of 0.110±0.014 *μ*M and 0.046±0.003 *μ*M, respectively (Figure 5c).

### MPT0B271 inhibits constitutive STAT3 activation in A549 cells

Next, we investigated the effect of MPT0B271 on the modulation of constitutive STAT-3 phosphorylation in A549 cells by western blot analysis using an antibody that recognizes phosphorylation at Tyr705. As shown in Figure 5d, MPT0B271 significantly inhibited the constitutive phosphorylation of STAT3 in a time-dependent manner. The level of STAT3 tyrosine phosphorylation declined dramatically in MPT0B271-treated A549 cells, as determined by the PathScan Phospho-STAT3 (Tyr705) Sandwich ELISA kit (Figure 5e). However, transfection with constitutively active STAT3 only slightly rescued cell viability in A549 cells (Figure 5f).

### MPT0B271 in combination with erlotinib increased tumor cell growth inhibition *in vitro* and *in vivo*

**Figure 6:**
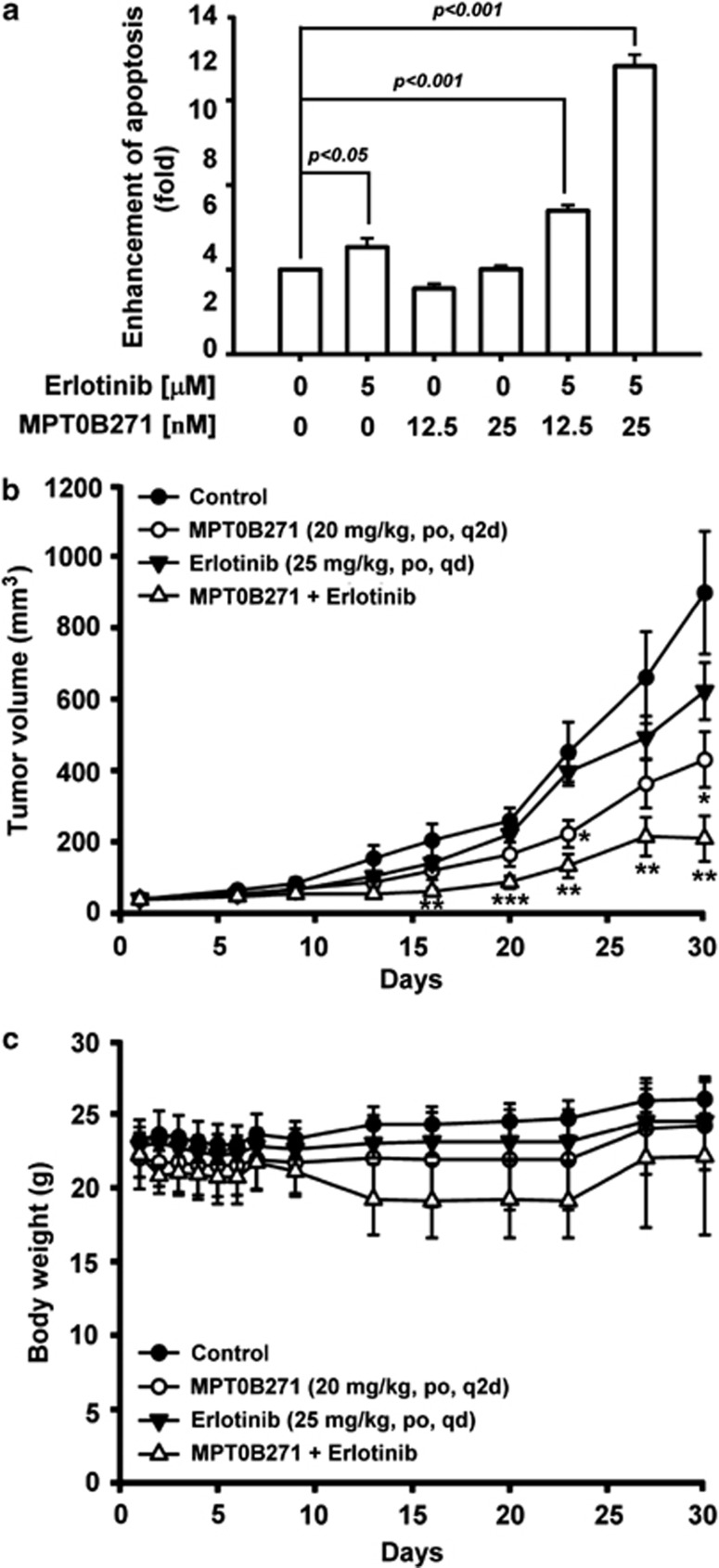
*In vitro* and *in vivo* antitumor activity of MPT0B271 in combination with erlotinib. (**a**) A549 cells were treated with erlotinib (5 *μ*M) in combination with MPT0B271 (0.0125 or 0.025 *μ*M) for 48 h, and cell apoptosis was measured using the Cell Death ELISA^PLUS^ kit. Data are expressed as the mean±S.E. of at least three independent determinations. (**b**) A549 xenograft model. A549-tumor-bearing nude mice were treated with vehicle, MPT0B271 (20 mg/kg/day by oral gavage q2d), erlotinib (25 mg/kg/day by oral gavage once a day), or MPT0B271 in combination with erlotinib. Tumor was excised when the tumor size reached 1200 mm^3^. (**c**) The body weight of the mice measured daily during the first week and then at the days of administration. *P<0.05; **P<0.01, and ***P<0.001 as compared with the control group

Erlotinib has proven efficacy in advanced NSCLC, but resistance to erlotinib also occurs.^23^ Thus, we investigated whether the combination of erlotinib and MPT0B271 is effective against erlotinib-resistant human NSCLC A549 cells. We chose these concentrations from dose-response curves (data not shown). As shown in Figure 6a and Supplementary Figure 2, erlotinib (5 *μ*M) in combination with MPT0B271 (0.0125 or 0.025 *μ*M) resulted in significantly higher cell death than the monotherapeutic treatments, as measured using an enzyme immunoassay for histone-associated DNA fragments. Finally, treatment of A549 xenograft-bearing nude mice with a combination of erlotinib and MPT0B271 resulted in significantly decreased tumor progression without loss of their body weight as compared with either erlotinib or MPT0B271 monotherapy (Figures 6b and c). These data indicate that erlotinib in combination with MPT0B271 is effective against erlotinib-resistant NSCLC cell lines.

## Discussion

In the present study, MPT0B271 was identified as a microtubule-depolymerizing agent and exhibited significant antitumor activity against various cancer cell lines, as well as drug-resistant sublines. It has been illustrated that p53 serves as a key player in cellular response to various extracellular and intracellular stresses such as DNA damage, oncogenic action, and microtubule disruption.^25^ Our results showed that MPT0B271 induces the expression of p53 protein in a time-dependent manner, but the knockdown of p53 cannot prevent apoptosis in cells treated with MPT0B271. In addition, A549 (wild-type p53), H1299 (p53 null), and H226 (p53 mutant) exhibited similar IC_50_ values, indicating that the antitumor effect of MPT0B271 may not correlate with the p53 status. The traditional microtubule-targeting agents in clinical use, such as paclitaxel, require intravenous administration with a long-term remedial course, causing physical and mental suffering and reducing the patients' quality of life. In addition, they often lead to hypersensitivity reactions and neuropathy.^26, 27^ Here, it is noteworthy that MPT0B271 possesses oral availability, improves solubility, and is efficacious *in vivo* against NSCLC tumor xenografts.

Drug resistance is a serious problem that restricts the use of microtubule-targeting agents for clinical therapy. The MDR P-gp, a plasma membrane protein overexpressed in multidrug-resistant tumor cells, is a major obstacle to the success of chemotherapy. Efflux of drugs caused by MDR proteins augments the elimination of drugs from target cells and leads to drug resistance.^28^ Many microtubule-targeting agents are substrates of P-gp, and higher doses of these drugs are required to achieve adequate intracellular concentrations in multidrug-resistant cancer cells.^8^ We employed Rh-123 and calcein AM, both substrates of P-gp and multidrug resistance-associated protein (MRP), as probes to detect chemical compounds interacting with MDR proteins. The calcein generated from calcein AM by esterase in a viable cell emits strong green fluorescence.^24, 29, 30^ Our results demonstrated that the accumulation of Rh-123 or the intracellular fluorescence of Calcein is not increased in MPT0B271-treated NCI/ADR-RES cells. We also tested the effect of verapamil in combination with MPT0B271 using SRB assay (Supplementary Figure 1). The GI_50_ values of the combination of verapamil with MPT0B271 in NCI/ADR cells were 16 nM, which indicated that the antiproliferative effect of combination was similar with that of treatment with MPT0B271 alone. These results demonstrated that MPT0B271 may not be a substrate or modulator of the P-gp efflux pump.

Similar to other microtubule-targeting agents, MPT0B271 induced a concentration-dependent G2/M blockade, as indicated by flow cytometric analysis and expression of the MPM-2 epitope, a mitosis-specific marker. Another marker of the G2/M phase is cyclin B1, which is localized in the cytoplasm in G2 phase but moves rapidly into the nucleus at the beginning of mitosis.^31^ Western blot analysis showed that the protein level of cyclin B1 in the nuclear fraction of MPT0B271-treated cells was significantly enhanced compared with that of untreated cells, implying that cells treated with MPT0B271 were arrested in the mitotic phase. Cdc2/p34 is a cell-cycle kinase responsible for the regulation of G2 progression and G2/M transition in all eukaryotic cells. It has been reported that the activity of Cdc2/p34 kinase depends not only on its association with cyclin B1 but also on its phosphorylation state.^32^ We found that MPT0B271 markedly reduced p-Tyr15 Cdc2/p34 levels and increased p-Thr161 Cdc2/p34 levels and was associated with cyclin B1 upregulation, but had no effect on Cdc2/p34 expression. The PLK-1 and aurora kinase A and B are active during mitosis.^33^ PLK-1 is an early trigger of G2/M transition in mammalian cells and has been implicated in the regulation of different processes including mitotic entry, spindle formation, and cytokinesis. Aurora kinase A and B are required for centrosome separation, mitotic spindle assembly, chromosome biorientation, and cytokinesis during mitosis.^34, 35^ Our results showed that MPT0B271 enhances the expression of PLK-1 and aurora kinase A and B proteins in a time-dependent manner, supporting the notion that MPT0B271 induces M-phase arrest in A549 cells.

Our results showed that in addition to inducing cell-cycle arrests in mitosis, treatment with MPT0B271 is followed by a subsequent increase in the hypodiploid (apoptotic sub-G1 peak) population of the cell cycle. The presence of cytoplasmic histone-associated DNA fragments induced by MPT0B271 was also observed, providing additional evidence that MPT0B271 induces apoptosis in A549 cells. Cysteine proteases (caspases) have a critical role in apoptosis through the proteolysis of specific targets. Both the death receptor-mediated pathway and the mitochondrial-mediated pathway result in the activation of caspase-3, and subsequently lead to the cleavage of PARP.^36^ Our results showed that treatment with MPT0B271 results in a significant induction of caspase-3 and -7 activation, but fails to promote the cleavage of caspase-8 and -9 in A549 cells. Caspases-8 and -9 probably have a minor role in apoptosis in A549 cells. Mcl-1 is required to prevent apoptosis induced by intrinsic and extrinsic pathways.^37^ Treatment with MPT0B271 reduced the expression of Mcl-1 in A549 cells. In addition, cell apoptosis was rescued by exogenous Mcl-1 overexpression, which signifies the role of Mcl-1 degradation in MPT0B271-induced cell apoptosis.

STAT3 is constitutively activated in most tumor cells and persistent STAT3 activation has been associated with both chemoresistance and radioresistance. The acetyl STAT3 translocates into the nucleus where it promotes cell proliferation and survival through transactivation of related genes. In addition, STAT3 can also associate with microtubules and mitochondria and regulate cell behavior. It was found that microtubule-targeting agents decrease the tyrosine phosphorylation-induced activation of STAT3 (Tyr705) in tumor cells, inhibit the expression of STAT3 target genes, and correlate with its cytotoxic effect.^38, 39, 40^ In our study, we found that MPT0B271 specifically reduced STAT3 phosphorylation at Tyr705. In A549 cells transfected with the STAT3-C plasmid, a minor reversal of cell cytotoxicity was observed, suggesting that STAT3 may have a minor role in MPT0B271-induced apoptosis in A549 cells and that other mechanisms may be involved in this phenomenon.

The overexpression of EGFR has previously been reported in a wide range of human malignancies including NSCLC and is a factor that is indicative of poor prognosis. The oral EGFR tyrosine kinase inhibitor Erlotinib (Terceva, Roche, Mannheim, Germany) reversibly binds to the intracellular domain of EGFR and blocks autophosphorylation of EGFR with subsequent suppression of the downstream signaling pathways that causes uncontrolled tumor cell growth and proliferation. Although erlotinib has proven efficacy in metastatic NSCLC and has been reported to confer a survival benefit for advanced NSCLC patients harboring EGFR mutations, resistance to erlotinib also occurs and reduces its efficacy.^41, 42, 43^ To overcome the problem of resistance, we combined the microtubule-binding agent MPT0B271 with erlotinib to increase the antitumor effects in the erlotinib-resistant human NSCLC cell line A549. Our data showed that erlotinib monotherapy did not result in significant cell death in A549 cells as compared with the control group, which was assessed using a cell death detection ELISA assay kit. However, combination therapy of MPT0B271 with erlotinib showed a synergistic effect in A549 cells. A similar result was obtained in the A549 subcutaneous xenograft mouse model. Erlotinib had no effect on tumor progression, but in comparison with MPT0B271 could produce a stronger antitumor effect. These results may indicate that MPT0B271 in combination with erlotinib would be a useful treatment in cases of NSCLC, which have developed progressive disease.

Taken together, our results indicated that MPT0B271 could be an effective orally administered microtubule-destabilizing agent with poor susceptibility to P-gp that possesses potent cytotoxic activity. We demonstrated that MPT0B271 inhibits tubulin polymerization, leading to mitotic arrest of the cell cycle and subsequently triggering apoptotic signaling pathways in human NSCLC cells. In addition, the antitumor growth effect of MPT0B271 in combination with the EGFR inhibition effect of erlotinib is more potent than the same drug used alone in NSCLC cells *in vitro* and *in vivo*. These findings suggest that MPT0B271 may foster novel therapeutic strategies for NSCLC.

## Materials and Methods

### Reagents

SRB (230162; Sigma-Aldrich, St. Louis, MO, USA), MTT (M2128; Sigma-Aldrich), PI (P4170; Sigma-Aldrich), FITC-conjugated anti-mouse IgG (F9295; Sigma-Aldrich), and all other chemical reagents were obtained from Sigma (St. Louis, MO, USA). 4',6-diamidino-2-phenylindole (DAPI) was purchased from Roche Molecular Biochemicals (Cat. 10236276001, Mannheim, Germany). Antibodies against cdc2 (Thr161) (Cat. 9114), cdc2 (Tyr15) (Cat. 4539), Aurora A (Cat. 4718), Aurora B (Cat. 3094), PLK1 (Cat. 4513), p53 (Sser15) (Cat. 2527), Bid (Cat. 2002), caspase-8 (Cat. 9746), and caspase-9 (Cat. 9502) were purchased from Cell Signaling Technology (Beverly, MA, USA). Antibodies against cyclin B1 (Cat. sc-594), cdc25C (Cat. sc-13138), cdc2 (Cat. sc-54), Bcl-2 (Cat. sc-7382), Bcl-xL (Cat. sc-8392), Bax (Cat. sc-7480), Mcl-1 (Cat. sc-819), Bak (Cat. sc-832), and PARP (Cat. sc-7150) were purchased from Santa Cruz Biotechnology (Santa Cruz, CA, USA). The antibody against caspase-3 (Cat. IMG-144A) was purchased from Imgenex (San Diego, CA, USA), and the antibody to MPM2 (Ser161/Thr97) (Cat. 05-368) was obtained from Upstate Biotechnology Inc. (Temecula, CA, USA). Antibodies to STAT3 (Cat. 610189), p53 (Cat. 554166), and caspase-7 (Cat. 556541) were purchased from BD Biosciences (San Jose, CA, USA). The antibody to STAT3 (Tyr705) (Cat. 2236-1) was obtained from Epitomics (Burlingame, CA, USA), and the antibody to actin (Cat. MAB1501) was purchased from Millipore (Temecula, CA, USA). The p53 si RNA (Cat. 106141) was obtained from Invitrogen (Carlsbad, CA, USA).

### Cell culture

The human NSCLC cell lines A549, NCI-H1299, and NCI-H226; the human pancreatic adenocarcinoma cell line AsPC-1; the human colorectal cancer cell line HCT-116; the human hepatoma cell line Hep3B; the human breast carcinoma cell line MDA-MB-231; the human prostate cancer cell line PC-3; the human ovarian carcinoma cell line SKOV3; and the human promyelocytic leukemia cell line HL60 were obtained from American Type Culture Collection (ATCC) (Manassas, VA, USA). The NCI/ADR-RES cell line was obtained from the DTP Human Tumor Cell Line Screen (Developmental Therapeutics Program, NCI). Cells were maintained in RPMI-1640 medium or DMEM with 10% fetal bovine serum (FBS) and penicillin (100 U/ml)/streptomycin (100 *μ*g/ml)/amphotericin (0.25 *μ*g/ml) at 37°C in a humidified incubator with 5% CO_2_.

### *In vitro* tubulin polymerization assay

The effect of identified compounds on tubulin polymerization was determined kinetically using the CytoDYNAMIX Screen kit (BK006P, Cytoskeleton Inc., Denver, CO, USA). Cold porcine tubulin protein (>99% purity) was added to G-PEM buffer (80 mM PIPES, 2 mM MgCl_2_, 0.5 mM EGTA, 1 mM GTP, pH 6.9) containing 15% glycerol with or without the identified compounds. The sample mixture was dotted onto a prewarmed 96-well plate, which was immediately transferred to a 37°C plate reader (SpectraMax Plus, Molecular Devices Inc., Sunnyvale, CA, USA). The absorbance was read every minute for 30 min at 340 nm.

### Immunofluorescence confocal microscopy

A549 cells were seeded sparsely in eight-well chamber slides and treated with or without identified compounds for 24 h. Following treatment, cells were fixed with cold methanol at −20°C for 15 min, washed three times with phosphate-buffered saline (PBS) and blocked with 1% PBS plus 0.1% Triton X-100 for 30 min at 37°C. Microtubules were detected by incubation with a monoclonal anti-*β*-tubulin at 37°C for 1 h. Then, the cells were washed with PBS and incubated with an FITC-conjugated anti-mouse IgG antibody. Nuclei were stained with DAPI, and microtubule distribution images were acquired with a Leica TCS SP2 Confocal Spectral Microscope (Leica, Wetzlar, Germany).

### Pharmacokinetic analysis

Male CD-1 (*Crl.*) mice (*n*=4 per group) were obtained from Lasco (Taipei, Taiwan) and used to examine the pharmacokinetics (PK) of the MPT0B271 compound. MPT0B271 was dissolved in polyethylene glycol 400/ethanol/water (60/5/35, *v/v*) and administered by a single intravenous tail vein injection at 2.0 mg/kg. For oral dosing, MPT0B271 was suspended in 0.5% methylcellulose (MC)/water and given via oral gavage with a dosing volume of 0.26 ml per animal (10 mg/kg). The animals were fasted for 16 h before dosing and allowed to consume standard chow 4 h post dosing. For mice treated via tail vein injection, plasma samples were collected before dosing and then 2, 5, 15, and 30 min and 1, 1.5, 2, 4, 6, 9, 24, and 27 h after dosing. For mice treated via oral administration, blood samples were collected before dosing and then 15 and 30 min and 1, 1.5, 2, 4, 6, 9, 24, and 27 h after dosing. The plasma concentrations of MPT0B271 were measured by LC-MS/MS (API4000; PE Sciex, Concord, ON, Canada) with a reverse-phase ODS column. The PK parameters were calculated from the mean plasma concentrations with the WinNonlin Professional program (version 5.2, Pharsight Corp., Mountain View, CA, USA).

### SRB assay

All cells were seeded in 96-well culture plates at a density of 3–5 × 10^3^ cells/well. After attachment, cells were fixed with 10% trichloroacetic acid (TCA) to provide a measurement of the cell population at the time of drug addition. The following day, cells were treated with vehicle (0.1% DMSO) or an increasing gradient concentration of the indicated compounds for 48 h, after which cells were fixed with 10% TCA and stained with 0.4% (w/v) SRB dissolved in 1% acetic acid. The protein-bound dye was subsequently extracted with 10 mM trizma base to determine the absorbance at a wavelength of 515 nm. The inhibition rate on cell proliferation (GI_50_) as a function of test drug concentration was calculated for each well as 100−[(A515_treated cells_−A515_time_)].

### MTT assay

Cells were incubated in the absence or presence of MPT0B271 for 48 h and then incubated with 0.5 mg/ml MTT solution for 1 h at 37°C. At the end of the incubation, the purple formazan crystals were dissolved in 100 *μ*l DMSO, and the absorbance was measured at 550 nm using an ELISA reader. The percentage of cell survival was plotted as a percentage to determine the IC_50_ values (50% growth inhibition).

### P-gp activity assay

Rh-123 and a Multi-Drug Resistance Assay kit (Calcein AM, Cayman Chemical, Ann Arbor, MI, USA), both of which include fluorescent substrates of P-gp, were used to measure the transport activity of P-gp. For the Rh-123 assay, NCI/ADR-RES cells were pretreated with or without the indicated compounds in culture medium in the dark at 37°C for 1 h and then co-treated with Rh-123 for an additional hour. After Rh-123 accumulation, the cells were washed with ice-cold PBS and collected by trypsinization. The intracellular fluorescence of Rh-123 was measured using a FACScan flow cytometer (BD Biosciences). The calcein AM efflux assay was performed according to the manufacturer's protocol. In brief, NCI/ADR-RES cells were seeded in 96-well culture plates at a density of 5 × 10^4^ cells/well and then treated with or without the experimental compounds. Calcein AM was added to each well, and the fluorescence was measured at an excitation wavelength of 485 nm and an emission wavelength of 535 nm.

### Cell-cycle analysis

Following treatment, the cells were harvested by trypsinization and centrifuged. The pellets were fixed with 70% (v/v) ethanol at −20°C overnight. The cells were again centrifuged at 2000 r.p.m. for 3 min, and the supernatant was discarded. Pellets were washed once with ice-cold PBS and resuspended in 0.1 ml phosphate/citric acid buffer (0.2 M Na_2_HPO_4_, 0.1 M citric acid, pH 7.8) for 30 min at room temperature. After incubation, the cells were stained with a PI working solution (0.1% Triton X-100, 100 *μ*g/ml RNase and 80 *μ*g/ml PI in PBS) in the dark. The fluorescence of the samples was measured with a FACScan flow cytometer with the CellQuest software (Becton Dickinson, San Jose, CA, USA).

### Nuclear extracts and western blot analysis

Cytoplasmic and nuclear protein extraction was performed as described previously.^44^ For western blot analysis, cell lysates were prepared, and proteins were separated by 7.5–15% SDS-PAGE, transferred onto PVDF membrane, and then immunoblotted with specific antibodies. Proteins were visualized with an ECL detection system (GE Healthcare Bio-Sciences, Pittsburgh, PA, USA).

### Evaluation of apoptosis

Quantitative oligonucleosomal DNA fragmentation was used to determine the ability of the identified compound to induce apoptosis. The Cell Death Detection ELISA^PLUS^ kit (Roche) was used according to the manufacturer's protocol.

### STAT3 kit

The level of STAT3 phosphorylation in response to MPT0B271 treatment was determined using the PathScan Phospho-Stat3 (Try705) Sandwich ELISA kit (Cell Signaling Technology) according to the manufacturer's instructions. The absorbance was read within 30 min at 405 nm using a microplate ELISA reader.

### Transient transfection

The siRNA against p53 and the negative control were purchased from Invitrogen. The Mcl-1 (25375) and STAT3-C plasmids were purchased from Addgene (Cambridge, MA, USA). A549 cells were grown to 70% confluence in a culture dish, and transfection was performed using Lipofectamine reagent (Invitrogen) according to the manufacturer's instructions. Following transfection, cells were allowed to recover for 24 h, seeded in 96-well or 6-well plates and then treated for another 48 h.

### A549 xenograft models

A549 cells (2 × 10^6^ cells/mice) in 0.2 ml culture medium were inoculated subcutaneously (sc) in *nu/nu* mice (male, 5–6 weeks). Once the tumor size was ∼100 mm^3^, mice were allocated at random to treatment groups: vehicle (0.5% CMC/0.1% Tween-80 in ddH_2_O); 5 or 10 mg/kg of MPT0B271 once a day (qd) or 20 mg/kg once every 2 days (q2d) by oral gavage; 25 mg/kg of erlotinib qd; or 25 mg/kg of erlotinib qd in combination with 20 mg/kg of MPT0B271 q2d. Caliper measurements were used to calculate tumor volume (*V*, mm^3^) using the formula *V*=*lw*^2^/2, with *l* being the length and the *w* being the width of the tumor. All animal studies were conducted in accordance with the guidelines of the Animal Care and Use Committee at National Taiwan University.

### Statistical analysis

Results are expressed as the mean±S.E. for the indicated number of separate experiments. Means were assessed for significant differences using *t*-test and *P*-values<0.05 were considered as significant.

## Abbreviations

P-gp: P-glycoprotein
Rh-123: rhodamine-123
calcein AM: calcein acetoxymethyl ester
PARP: poly (ADP-ribose) polymerase
MDR: multidrug resistance
MRP: multidrug resistance-associated protein
STAT3: signal transducer and activator of transcription 3
EGFR: epidermal growth factor receptor
NSCLC: non-small cell lung cancer
PLK1: serine/threonine kinases polo-like kinase 1
DAPI: 4,6-diamidino-2-phenylindole
PI: propidium iodide
siRNA: small interfering RNA
