# Supplementary figures and images for: Orally active microtubule-targeting agent, MPT0B271, for the treatment of human non-small cell lung cancer, alone and in combination with erlotinib
Source: Cell Death Dis. 2014 Apr 10;5(4):e1162–. doi: 10.1038/cddis.2014.128 (PMC5424107; doi:10.1038/cddis.2014.128)

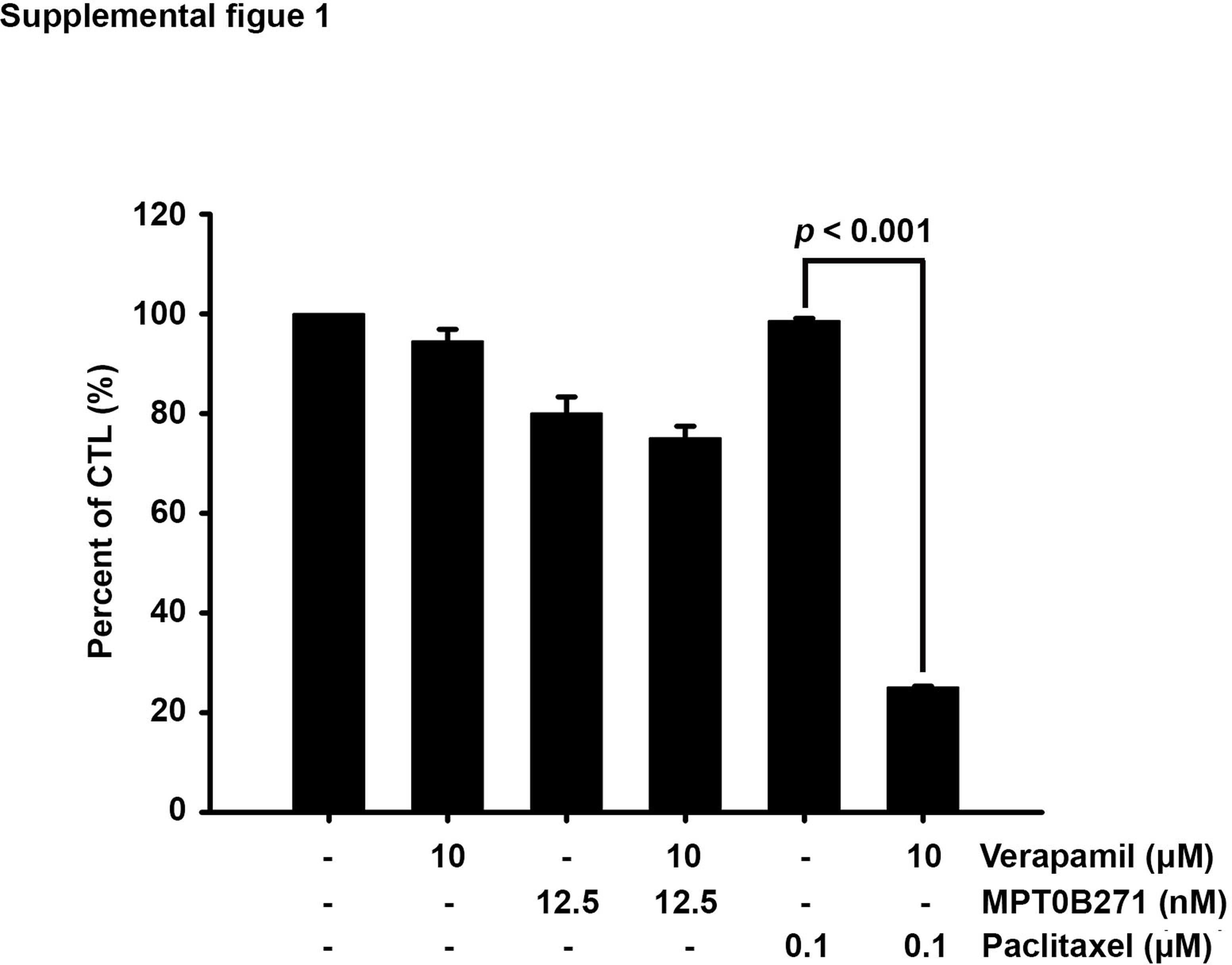

Supplement: Supplementary Figure 1 [file cddis2014128x1.tif]

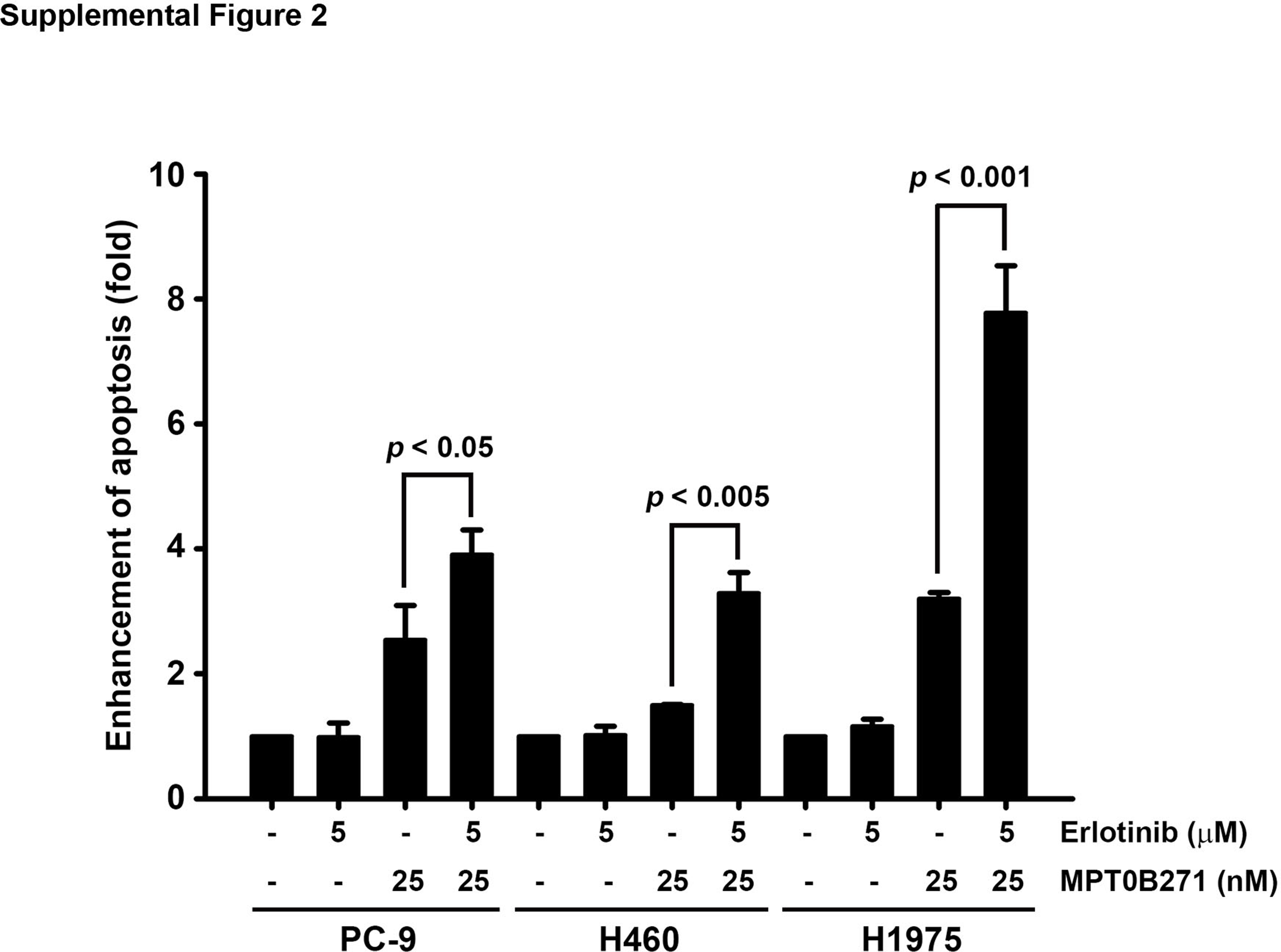

Supplement: Supplementary Figure 2 [file cddis2014128x2.tif]
