# Supplementary material for: Orally active microtubule-targeting agent, MPT0B271, for the treatment of human non-small cell lung cancer, alone and in combination with erlotinib
Source: Cell Death Dis. 2014 Apr 10;5(4):e1162–. doi: 10.1038/cddis.2014.128 (PMC5424107; doi:10.1038/cddis.2014.128)
Supplement: Supplementary Figure Legends [file cddis2014128x3.doc]

**SUPPLEMENTARY FIGURE LEGEND**

Supplemental figure 1. Antiproliferative effect of MPT0B271 in combination with erlotinib in NCI/ADR cells using SRB assay.

NCI/ADR cells were treated with or without verapamil, MPT0B271 or paclitaxel at indicated concentrations for 48 hours. And then cells were fixed and stained with SRB. The protein-bound dye was subsequently extracted with 10 mM trizma base to determine the absorbance at a wavelength of 515 nm.

Supplemental figure 2. Apoptotic effects of MPT0B271 in combination with erlotinib in several human non-small cell lung cancer cells.

PC-9, H460 and H1975 NSCLC cells were treated with or without erlotinib, MPT0B271, and combination of MPT0B271 and erlotinib for 24 hours. Cell apooptosis was analyzed by Cell Death Detection ELISA^PLUS^ kit (Roche Diagnostics).
